# Hidden Markov Models: The Best Models for Forager Movements?

**DOI:** 10.1371/journal.pone.0071246

**Published:** 2013-08-23

**Authors:** Rocio Joo, Sophie Bertrand, Jorge Tam, Ronan Fablet

**Affiliations:** 1 Unité Mixte de Recherche (UMR) 212 Exploited Marine Ecosystems, Institut de Recherche pour le Développement (IRD), Sète, France; 2 Instituto del Mar del Perú, Callao, Perú; 3 UMR 6285 Information and Communication Science and Technology Laboratory (LabSTICC), Institut TELECOM/TELECOM Bretagne, Brest, France; Cajal Institute, Consejo Superior de Investigaciones Científicas, Spain

## Abstract

One major challenge in the emerging field of movement ecology is the inference of behavioural modes from movement patterns. This has been mainly addressed through Hidden Markov models (HMMs). We propose here to evaluate two sets of alternative and state-of-the-art modelling approaches. First, we consider hidden semi-Markov models (HSMMs). They may better represent the behavioural dynamics of foragers since they explicitly model the duration of the behavioural modes. Second, we consider discriminative models which state the inference of behavioural modes as a classification issue, and may take better advantage of multivariate and non linear combinations of movement pattern descriptors. For this work, we use a dataset of >200 trips from human foragers, Peruvian fishermen targeting anchovy. Their movements were recorded through a Vessel Monitoring System (∼1 record per hour), while their behavioural modes (fishing, searching and cruising) were reported by on-board observers. We compare the efficiency of hidden Markov, hidden semi-Markov, and three discriminative models (random forests, artificial neural networks and support vector machines) for inferring the fishermen behavioural modes, using a cross-validation procedure. HSMMs show the highest accuracy (80%), significantly outperforming HMMs and discriminative models. Simulations show that data with higher temporal resolution, HSMMs reach nearly 100% of accuracy. Our results demonstrate to what extent the sequential nature of movement is critical for accurately inferring behavioural modes from a trajectory and we strongly recommend the use of HSMMs for such purpose. In addition, this work opens perspectives on the use of hybrid HSMM-discriminative models, where a discriminative setting for the observation process of HSMMs could greatly improve inference performance.

## Introduction

Movement paths result from the interaction between the behaviour of an organism and the spatial structuring patterns of its environment [1]–[5]. Those paths result from the succession of distinct types of behavioural modes (e.g., travelling from one area to another, searching for cues or preys, pursuing and eating a prey), each one associated with the fulfilment of a particular goal. The knowledge of these modes provides rich information on the processes underlying movement, but they are not directly accessible through the sole observation of the sequence of positions recorded by GPS or other position-logging artefacts. The inference of the behavioural modes from movement paths remains a challenging issue in the emerging field of movement ecology [6].

Hidden Markov models (HMMs) have become increasingly popular to address this issue (for examples in classifying activities such as foraging, searching, encamping, cruising, migrating and bedding, see [7]–[18]; in navigation strategies, see [19]–[21]; and in types of movement orientation, see [22]). HMMs rely on probabilistic inference of the behavioural modes, stated as hidden states, from the *in situ* observed series. Those series are typically sequences of positions or associated features such as distances, speeds or turning angles along the movement paths [8]. The key feature of HMMs is to account for the temporal dynamics of the behavioural modes, mostly based on state transitions between steps (two consecutive positions define a step). Such first-order HMMs comprise computationally efficient inference procedures [23], [24]. However, it may be unrealistic to consider that a forager takes a decision about changing its behavioural mode at each step, and regardless of any behaviour dating from more than one step back. In this respect, hidden semi-Markov models (HSMMs), recently investigated in movement ecology [8], may be more appealing. While HMMs characterize behaviour at the step scale, HSMMs characterize behaviour at the segment scale; a segment is composed of consecutive steps associated with a same state. HSMMs do account for transitions between consecutive but distinct states and for durations of state segments corresponding to one behavioural mode.

For most living organisms studied in ecology, groundtruthed datasets – samples of tracks or positions for which behavioural modes are known – are hardly available. Therefore, inference issues are generally stated within a non-supervised framework. Furthermore, rigorous model validation (e.g. by cross-validation as in [25], [26]) cannot be performed. Model validation mainly relies on some expert-driven evaluation of the ecological or behavioural plausibility of the behavioural modes inferred. Fishermen had long been the only foragers whose true behavioural modes were available. Actually, on-board observers can provide direct observations of the vessels’ activities during fishing trips, allowing for model validation [11]. In pelagic ecosystems, water masses and fish schools are constantly moving [27] so that precise prey localization is unpredictable regardless of the predator, human or animal [28]. Foraging movements for all those predators aim at the same goal, i.e. dealing with uncertainty on prey localization and maximizing prey encounters. As a result, fishermen deploy similar foraging strategies to those of other animal predators [28], therefore the same statistical methods used for other foragers are applied to them (for instance, see [28], [29] for a characterization on their diffusive movement through Lévy walks; and see [10]–[12] for applications of HMMs for identifying their behaviour). Nowadays, tracking data on non-human foragers can be enriched by concurrent deployment of additional devices that explicitly record activities, at least for a sample of individuals (e.g. time-depth recorders for diving as in [9], [30]–[32], video-cameras as in [33], [34] and tri-axial accelerometers as in [35]). Given such partially groundtruthed datasets, we are no more in a fully non-supervised context, but rather in supervised or semi-supervised ones [36], [37].

Markovian models may apply in such supervised setting. Nevertheless, alternative models may also be considered, particularly because Markovian models are limited for handling multiple observed variables. Choosing and fitting the most appropriate multivariate distribution may be delicate. A simplifying hypothesis is commonly adopted to solve this issue: observed variables conditioned on states are assumed mutually independent, so the multivariate distribution becomes the product of the univariate conditional distributions of each variable. By contrast, discriminative models, such as random forests (RFs), artificial neural networks (ANNs) and support vector machines (SVMs), provide robust solutions for non-linear discrimination in high-dimensional spaces. They have been shown to be highly efficient for a wide range of applications [38]–[42]. Their availability in several software without the need of strong computational skills makes them attractive for applications to ecological datasets [43], [44]. This includes a few studies dedicated to behavioural modes [45]–[47]. This context of technological advances for data collection enables a wide range of supervised models. Hence, evaluating and comparing models accuracy for inferring behavioural modes becomes necessary.

Here, we consider as study case the foraging movement of 50 Peruvian purse-seiners targeting anchovy. More than 200 of their fishing trips were documented by a Vessel Monitoring System and their behavioural modes simultaneously registered by on-board observers. This unique and large groundtruthed dataset allows performing, via cross-validation, a comprehensive evaluation and comparison of Markovian (HMMs and HSMMs) and discriminative models (random forests, artificial neural networks and support vector machines) for inferring the behavioural modes of a moving forager. We show that HSMMs provide the most accurate inference of the behavioural modes with 80% of global accuracy. We also show via simulation that this result could be greatly reinforced with position records of higher frequency.

## Materials and Methods

The purse-seine Peruvian anchovy fishery is the world largest mono specific fishery [48]. Satellite tracking by Vessel Monitoring System (VMS) is mandatory for the whole Peruvian industrial fishing fleet (>1000 vessels) since 2000. Vessel positions (±100m of accuracy; ∼1 record per hour) for tens of thousands of fishing trips are thus available for scientific purposes since (e.g., [28]–[30], [46], [47], [49]). Although most records are given according to one-hour intervals, some irregularities (e.g. 0.17, 0.99, 12) seldom occur. Since there is no straightforward optimal interpolation method for these cases [8], we work with the records as they are. Therefore, the considered VMS data consist in tracks (i.e., series of positions) with non-regular steps. For each VMS track, several observed variables are computed at each step: speed (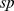), heading (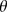), changes of speed and turning angles between the previous and the current step (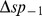 and 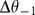) and between the current and the next step (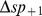 and 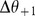).

In addition, IMARPE (Peruvian Marine Research Institute) runs a program of observers on-board for a ∼1% sample of the fishing trips. They record the location and time of the different behavioural modes occurring during the trips: fishing, searching, cruising (i.e. travelling following a predetermined course), drifting, helping other vessels, and receiving or giving fish to other vessel. For the remaining 99% of the fishing trips, behavioural modes are unknown.

**Figure 1:**
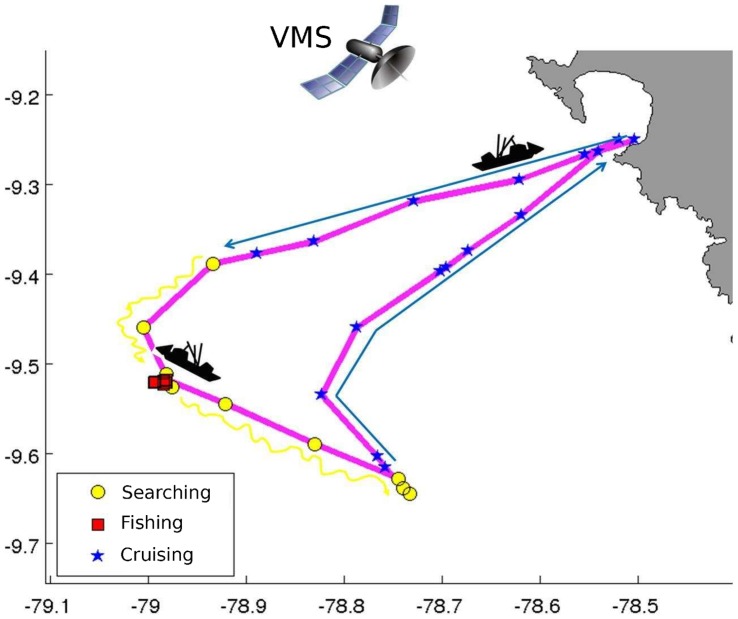
Fishing trip with VMS records and their corresponding behavioural modes.

Based on the criteria described in [28], [29], [47], a groundtruthed dataset gathering tracking data and their corresponding behavioural modes is built. Overall we consider three behavioural modes, fishing, searching and cruising. Fishing trips involving ‘helping’, ‘receiving/giving’ and ‘drifting’ modes are discarded, due to the low number of occurrences of these modes. Together they represent less than 6% of the groundtruthed dataset. We work with a dataset corresponding to 2008, consisting of 242 fishing trips (∼36000 fishing trips were performed in total that year). Fig. 1 shows an example of a trip with each VMS record associated with a behavioural mode.

For hidden state inference, two different approaches are investigated and evaluated. Markovian models, which take into account the sequential nature of data; and discriminative models, remarkably popular in the pattern recognition and machine learning domain [50]–[52]. Henceforth, we will denote by 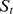 the state variable at time 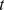 taking a discrete value 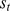, which encodes a behavioural mode (fishing, searching or cruising). A state sequence starting at time 0 and ending at 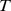 is then denoted by 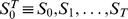, taking discrete values 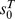. Likewise, 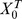 represents the sequence of continuous observed variables taking values 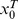. Under the two approaches, the goal is to infer 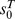.

We perform a quantitative evaluation of the models performance using a classic cross-validation procedure. It proceeds as follows. The groundtruthed dataset is split into two sub-samples. The first partition is used for training the models, i.e., learning from the data and estimating the parameters. The second partition is used for validating the models, i.e., evaluating model performance. Training and validation partitions gather each 50% of the original sample of trips and are built by repeated random sub-sampling (20 repetitions). This parameter setting provides us with a trade-off between the performance evaluation and computational efficiency.

### Markovian Models

#### HMM

HMMs are the classic models for inferring hidden state sequences from observed variables [53]. A HMM combines the two following processes. An underlying first-order Markov process of the hidden state sequence, where the probability of currently being at state 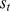 only depends on the immediately preceding state 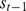. And a state-dependent observation process, where the probability of 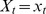 only depends on the current state 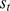 and not on previous states or observations. Assuming homogeneity, a HMM can be fully characterized by (1) the initial probabilities 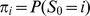, (2) the transition probabilities 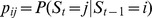, and (3) the state-dependent observation probability density functions (pdfs) 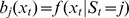, where 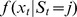 denotes the conditional pdf of 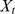 at 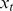 given 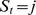. When observations are multivariate, under mutual independence
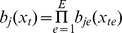
where 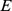 is the number of observed variables included in the model. The likelihood of a HMM can be written as



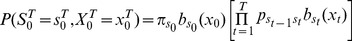



In our case study, several observed variables are available (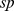,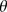,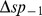,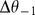,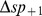 and 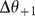). Over all possible combinations of observed variables, the subset (combination) of variables giving the highest state-inference accuracy is chosen –the computation of accuracy as well as other performance indicators are described in section ‘Indicators of model performance’. For each observed variable, we test several probability distributions based on a supervised maximum likelihood (ML) fit. When ML estimation cannot be derived analytically, a numerical optimization is used. Goodness-of-fit (GOF) is tested using the robust Cramér-von Mises statistic [54]. In cases where two or more distributions provided significant fits, the AIC criterion [55] is used for selection among them. All fishing trips start in cruising mode, so initial probabilities are set to one for cruising and zero for the other states. Given the training partition, the ML estimation of the transition probabilities resorts to computing the relative frequencies of the transitions between successive states [36]. Using all these elements, the inference of the sequence of hidden states 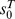 is done by global decoding via the Viterbi algorithm [23]. Hidden Markov Model toolbox for Matlab [56] is used.

#### HSMM

A first-order Markov state process may not be, however, the most natural choice for the interpretation of movement patterns. It implicitly assumes that time spent at a given state is distributed according to a geometric distribution. This distribution is memoryless; it means that at a given time 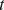, the waiting time for switching from one state to a distinct state is independent from the time already spent in the former state. However, in practice, a forager’s behaviour is not memoryless. A semi-Markov process may therefore be more suitable. It explicitly models the state duration distribution and may consider any distribution function. HSMMs are thus generalizations of HMMs. They combine two processes: a state-dependent observation process as in HMMs, and an underlying semi-Markov state process. A semi-Markov process is determined by the duration distributions 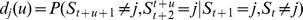 and transition probabilities between distinct states 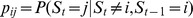. For the last visited state, a survival function of the duration is used: 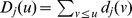. The likelihood of a HSMM [57] can be written as
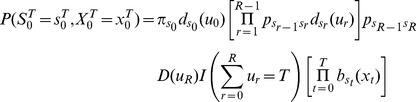
where 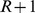 is the number of visited states, 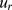 is the duration at state 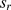, and 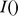 denotes the indicator function.

**Figure 2:**
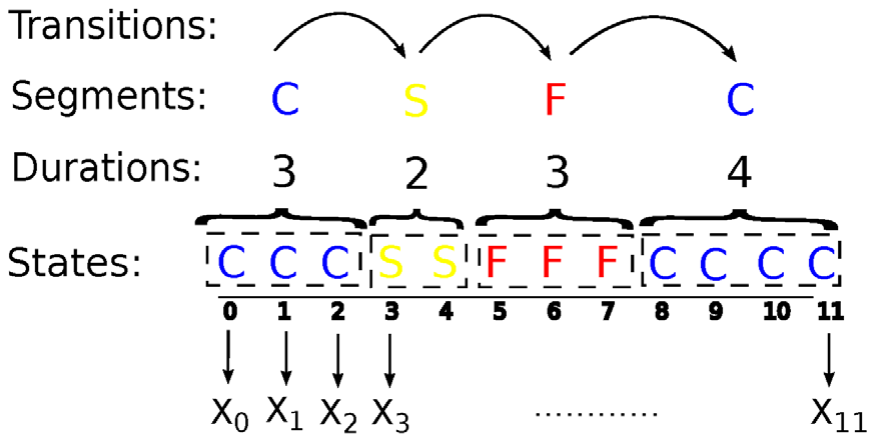
Schematic representation of a HSMM. At each step, an observed feature 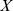 is related to a state, which encodes a behavioural mode (C: cruising, F: fishing, S: searching). The state process is modelled at the segment scale and it is characterized by durations and transitions as shown above.

Therefore, compared to HMMs, HSMMs provide a model of the state process at a higher scale: the segment scale (Fig. 2; [58], [59]). This segment scale is potentially more relevant for interpreting and discriminating distinct behavioural modes in foraging movement.

The selection of observed variables, the fit of state-dependent observed variable distributions and the estimation of transition probabilities (between distinct states) follow the same criteria as for HMMs. Although state durations are inherently discrete, continuous distributions provide flexibility under certain irregularities on the frequencies of positioning of satellite records. They enable the incorporation of those data directly into the model. Extensive literature on the use of continuous distributions for modelling duration is available (e.g., [58]–[61]). Here we examine seven continuous probability distributions for modelling the duration of each of the three behavioural modes. Their parameters are estimated by maximum likelihood using the training dataset. Then, GOF is tested using Cramér-von Mises statistic and AIC criterion is used for selection among distributions not rejected by the test. Using all these elements, the inference of the sequence of hidden states 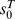 is done by global decoding via the forward-backward Viterbi algorithm [62]. A code in Matlab for this Viterbi algorithm is given in Text File S3.

### Discriminative Models

Discriminative models are alternative approaches for inferring behavioural states within recorded trajectories. In contrast to Markovian approaches, discriminative models do not rely on the explicit modelling of the joint likelihood of observation and state sequences. The inference of the behavioural mode sequence 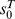 is stated as a classification issue, i.e. the determination of the class (behavioural mode) attached to any position along the trajectory. Within a supervised framework, discriminative models learn a classification rule to predict a class from an observed vector 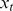. Random forests [63], support vector machines [64] and artificial neural networks [65] are among the state-of-the-art techniques in the machine learning domain [41]. These models differ in the way classification rules are stated and learned. For SVMs, the goal is to maximize the margin around the hyperplane that separates classes. For ANNs, the objective is to minimize the classification error. And for RFs, discrimination is achieved by the simultaneous minimization of the within-group variances and maximization of the between-group variances. The relative performances between these methods are application-dependent and vary according to the structure of the observation space [66]. A key feature of discriminative models is that they do not require any assumption on the nature of the observed variables, their distributions or covariances. To prevent over-fitting during the learning stage, a cross-validation procedure can be applied. Still, it requires sufficiently large and representative groundtruthed datasets.

As for HMMs and HSMMs, the subset of observed variables giving the highest inference accuracy is selected. The selected subsets may differ among the three discriminative models. Architecture and parametrization of each discriminative model is described below.

#### RFs

A random forest involves a set of 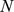 decision trees. A decision tree discriminates patterns recursively in a tree-like structure. At each tree node, 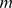 variables are randomly selected among the subset of observed variables. Data are split following certain conditions on those 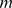 variables, so that within-group variance is minimized and between-group variance is maximized. For each observed vector 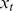, a tree’s output is its classification in a behavioural mode. Consequently, a random forest’s output is the statistical mode of the classification outputs of 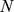 trees. We test 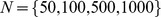 and 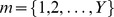, where 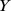 is the size of the subset of observed variables. The Matlab implementation of the random forest library [68] is used.

#### SVMs

Support vector machines are based on linear discrimination. A Gaussian kernel is used here for mapping the originally observed vectors into a new space in which classes (i.e., behavioural modes) may be linearly separated. Tested values for the scale parameter of the Gaussian kernel are 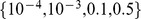. SVMs also involve a regularization parameter 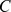. Increasing the value of 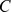 increases the cost of misclassifying points and decreases generalization power of the model. We test 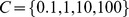. The Matlab™ implementation of the Libsvm library [68] is used.

#### ANNs

Multilayer perceptrons (MLPs) are the most widely used architectures of ANNs. Neurons are organized in layers. The first layer is composed of the observed variables and the last layer is composed of the model classification output. Between those first and last layers, one or more hidden layers can exist. Here, we use a MLP with one hidden layer as in [47]. Considered options for the number of hidden neurons range from one to ten. The Matlab neural network toolbox is used for the analysis.

For each discriminative model, we determine the optimal parameter setting according to the classification accuracy.

### Indicators of Model Performance

Overall, we aim at accurately reconstructing the sequence of states associated with each foraging trip. We consider two scales of analysis. First, we evaluate the accuracy of the inference at the step scale, and define the accuracy indicator as the percentage of individual steps where the inferred states correspond to the real ones. Second, we assess model performance at the segment scale (Fig. 2), which best characterizes behavioural modes. We use three indicators for each behavioural mode:

- The segment-level precision, defined as the percentage of inferred segments where the inferred behavioural mode corresponds to the true one.
- The segment-level recall, defined as the percentage of real segments where the true mode is correctly inferred.
- The F-measure or F1, which combines precision and recall performances [69]. It is defined as the harmonic mean of precision and recall, and reported here in terms of percentage similarly to precision and recall indicators.

Accuracy, precision, recall and F1 are standard performance evaluation measures in supervised contexts [70]. Beyond these performance measures, we also investigate the extent to which the considered models deliver a relevant global characterization of foraging patterns, particularly regarding the shape of the distributions of the behavioural mode durations. In this respect, we define a fourth indicator at the segment scale, called duration. This auxiliary indicator is computed as the mean squared difference between the empirical cumulative distribution functions of both real and inferred mode durations. Its values range from 0 to 1, where 0 refers to an error-free inference.

Formulas for the computation of all these indicators are shown in Table 1. Further details as well as an illustrative example on the computation of accuracy, precision, recall and F1 are described in Text File S1.

**Table 1:** Indicators of model performance.

Scale	Indicator
Step	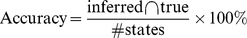
Segment	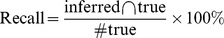
	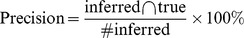
	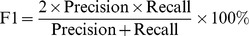
	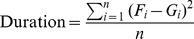

*Notes:* F and G represent empirical cumulative distributions for the real and inferred durations of a given behavioural mode, respectively.

## Results

The selected distributions for the state-dependent observation process (for HMMs and HSMMs) and for the duration of the states (for HSMMs) are shown in Table 2. For further details, AIC values of distributions with significant fits for observed variables and durations corresponding to the HSMM (Table 3) are indicated in Table S1.

**Table 2:** Distributions for each observed variable and duration conditioned on states.

Observed Variable	Searching	Fishing	Cruising
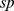	generalized Pareto	generalized extreme value	Gaussian mixture
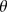	uniform	wrapped Cauchy	Laplace-Gaussian mixture
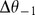	Kumaraswamy	uniform	loglogistic
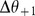	Beta	uniform	loglogistic
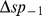	Laplace	Gaussian mixture	Student’s t
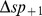	Laplace	Gumbel	Student’s t
Duration	generalized extreme value	lognormal	generalized extreme value

*Notes:* When Beta and Kumaraswamy distributions are used, data is transformed to scale from 0 to 1.

**Table 3:** Performance of all models for their corresponding best subsets of observed variables.

Mode	Model	HSMM	HMM	SVM	RF	ANN
	Subset	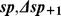	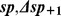	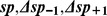	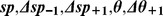	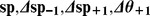
	Accuracy	**80.3%**	79.1%	79.0%	76.4%	79.2%
F	Recall	86.6%	84.7%	88.3%	85.5%	88.5%
	Precision	69.3%	69.4%	65.8%	64.5%	65.7%
	F1	**77.0%**	76.3%	75.4%	73.5%	75.4%
	Duration	4	14	17	29	22
S	Recall	37.5%	64.9%	56.3%	62.0%	59.5%
	Precision	56.9%	56.1%	57.6%	47.5%	54.3%
	F1	**66.7%**	60.2%	56.9%	53.7%	56.9%
	Duration	7	4	33	41	26
C	Recall	91.0%	87.4%	89.6%	76.9%	88.7%
	Precision	87.3%	86.4%	71.9%	72.0%	75.2%
	F1	**89.1%**	86.9%	79.8%	74.3%	82.1%
	Duration	1	2	7	25	7

*Notes:* In bold, the highest values of accuracy and F1. F: fishing; S: searching; C: cruising. Duration values are scaled by (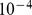).

For evaluating and comparing the two Markovian and the three discriminative models, we selected, for each model, the subset of observed variables which led to the greatest inference performance in terms of accuracy rate. Performance indicators at step and segment scales are reported for each of these models (Table 3). All models infer states with an accuracy greater than 75%. By a small though significant difference (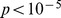 in paired-sample randomness tests; [71]), the HSMM’s accuracy is the highest.

Regarding behavioural modes, cruising seems to be the easiest mode to identify. All models show greater F1 scores for the cruising mode (between 74% and 89%). Likewise, the greatest recall and precision values correspond to cruising for all models. Relevant F1 scores are also reached for fishing mode inference (between 73% and 77%). By contrast, the identification of the searching mode appears difficult for all models (F1 between 54% and 67%). This behavioural mode involves relatively large confusion rates with both fishing and cruising modes (between 15% and 19% of the searching states are classified as fishing, and between 25% and 34% are classified as cruising, among all models).

For each behavioural mode, the HSMM outperforms all the other models (greatest F1 scores of 77%, 67% and 89% for fishing, searching and cruising, respectively). The second best model is the HMM. Differences between F1 scores of the HSMM and the HMM are significant for all behavioural modes (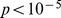 in all cases). Among the discriminative models, the ANN is the best model, followed closely by the SVM.

**Figure 3:**
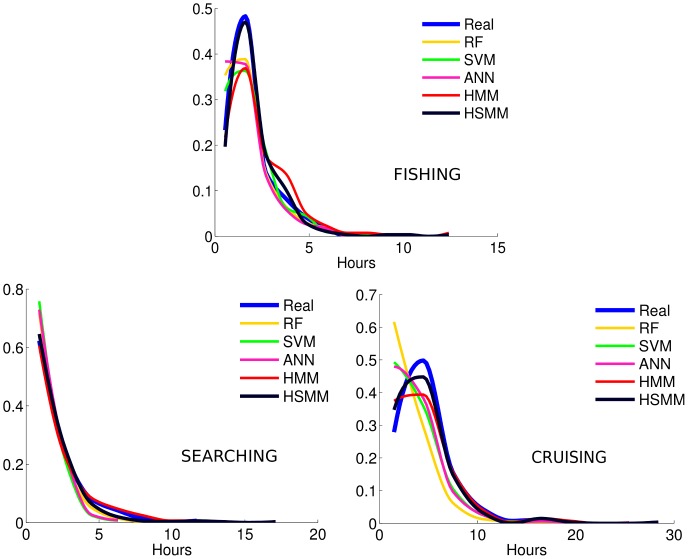
Distribution of the duration of each behavioural mode. For each model, an empirical distribution of the duration of each mode is estimated based on the duration of all inferred segments encoding the mode. RF: random forest. SVM: support vector machine. ANN: artificial neural network. HMM: hidden Markov model. HSMM: hidden semi-Markov model. Real: known behavioural modes.

The analysis of the distribution of the inferred durations for each behavioural mode leads to similar conclusions. In Fig. 3 it can be observed that all three discriminative models show higher empirical densities for low duration values than the Markovian models and the groundtruth. Discriminative models, i.e. RF, SVM and ANN, which do not consider state transitions nor durations, tend to under-estimate the duration of modes due to over-segmentation. By contrast, the Markovian models, particularly HSMM, provide more accurate estimates of these durations. Whereas the distribution of the durations for fishing and cruising modes are clearly better represented with the HSMM (duration statistics of 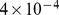 and 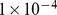 for fishing and cruising, respectively; Table 3), the HMM gives slightly better results for the searching mode (duration of 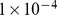).

**Figure 4:**
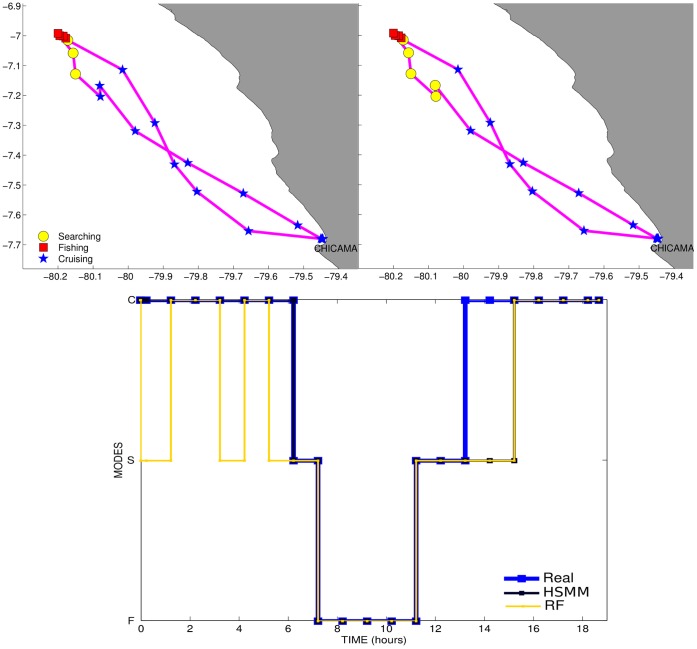
A fishing trajectory. Left upper panel: track with real behavioural modes. Right upper panel: track with inferred modes using the HSMM. Lower panel: temporal representation of the behavioural mode sequences, real and inferred, where 0 in the x-axis represents the beginning of the trip.

The over-segmentation problem is illustrated for one trajectory sample when comparing the sequences of behavioural modes inferred by the HSMM and the RF with the true sequence of modes (Fig. 4). There is strong over-segmentation in the sequences inferred by the RF, leading to under-estimation of the duration of the segments. By contrast, the HSMM achieves relevant representation of the mode sequences through time (Fig. 4, low panel) and thus also through space (Fig. 4, right panel).

Regarding computational cost, we compare all five models in Table 3 for one replica where 121 tracks were randomly selected for training and the remaining 121 for validation. The HMM shows the lowest computational time (16.78 seconds), followed by the RF and the SVM models (22.09 and 23.07 seconds, respectively). Next it is the HSMM (64.04 seconds) and finally the most expensive one is the ANN (140.14 seconds). For the HMM and the HSMM, the computational time comprised the estimation of the probability density function parameters and Viterbi algorithm application. For the SVM, the RF and the ANN, it comprised the optimal parameter setting, as described in the Methods section. The high computational cost of the ANN could be greatly affected by the call to a graphical interface as automatically performed by the Neural Network toolbox of Matlab™. This computational analysis should only be regarded in relative terms. Optimized implementations of these models could be expected to provide important computational gains (by a factor of 10 or more).

## Discussion

With a representative groundtruthed dataset composed of 242 fishing trips, we perform a comprehensive cross-validation evaluation of different Markovian and discriminative models for inferring behavioural modes from trajectory data. Our results show that the HSMM is the best model and enlighten several critical issues.

### State Dynamics are Key Information

Markovian models have the strength of considering the sequential nature of the data: state transitions are explicitly modelled and the sequence of states is inferred as the most likely sequence given the performed trajectory. However, they present limitations for incorporating the information contained in the observed variables, especially in cases of non-Gaussian multivariate observation spaces. Practical applications of Markovian models often involve simplifications such as independence and/or Gaussianity assumptions for modelling the multivariate distribution of the observed features given the behavioural modes. In contrast, discriminative models state the inference of behavioural modes as a classification issue. They use powerful non-linear and multivariate classification rules. At the step scale, the HSMM surpassed the discriminative models by small differences (+1% of accuracy with respect to the ANN and the SVM, and +4% with respecto to the RF; Table 3). At the segment scale, the surpassing outperformance of the HSMM was clearer (differences in F1 scores between +1.6% and +9.8% regarding both the ANN and the SVM, and between +3.5% and 14.8% regarding the RF; Table 3). This evidences that the information contained in the state sequence is key for accurately inferring the behavioural modes.

### HSMMs are Recommended for Behavioural Mode Inference

**Figure 5:**
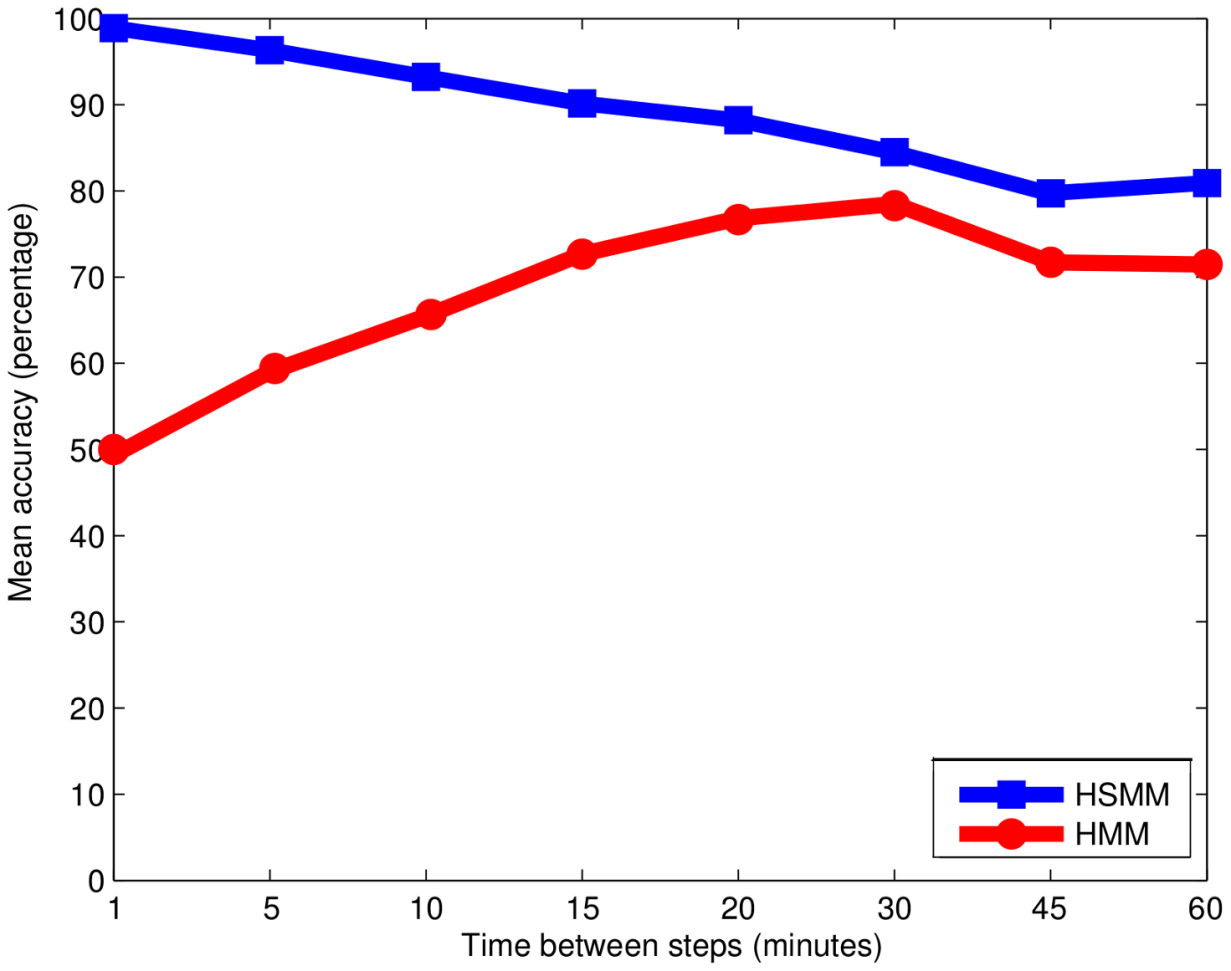
Mean accuracy for simulated sequences for different sampling rates using HSMM and HMM.

To our knowledge, our study presents the first application of HSMMs to foraging tracks using groundtruthed data on behavioural modes. For this study case, with steps of ∼1 hour, the HSMM performed slightly better than the HMM. A simulation study on high-resolution data (one-second steps) is described in Text File S2. We applied HSMMs and HMMs to sub-sampled versions of these sequences. The performance of each model was assessed by the mean accuracy (MA), which is the average of the accuracy for each behavioural mode (Fig. 5). For one-minute steps, the HMM performed very poorly, whereas for 30-minute steps it was by far more relevant (50% vs 78% of MA). By contrast, MA rates for the HSMM remained above 80% for all time steps. The HSMM actually benefited from high-resolution sequences – when available – to significantly improve inference performance (100% of MA for one-minute steps). These additional results clearly illustrate that the relevance of the first-order Markov state process embedded in HMMs greatly depends on the time steps of the trajectory data. By contrast, we show that the relevance of the HSMM does not decrease with smaller time steps. Likewise, [72] showed that reducing time steps severely decreased the performance of first-order Markov processes for estimating animal spatial distributions from tracking data.

Alternatively, higher-order (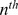-order) hidden Markov models account for additional complexity in the dynamics of the state sequence. They comprise a 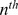 memory, i.e. the state value 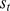 depends on the state values taken at the 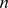 preceding states. They implicitly involve more general distributions on state segment durations than geometrical distributions. Therefore, they should outperform first-order HMMs for high-resolution sequences. However, in most practical problems the choice of the order of the hidden Markov model is not obvious and depends on both the time resolution of the data and the characteristic durations of the state segments. In addition, they are computationally expensive. HSMMs avoid the problem of choosing and fixing an order for the Markovian process. By considering transitions between distinct state segments and distributions on their durations, HSMMs model the scale of a homogeneous behavioural mode. By considering any distribution for modelling duration probability, HSMMs explicitly model the time an individual stays in a behavioural mode, rather than simply accepting the geometric decay of the duration distribution imposed by standard first-order HMMs [8]. Moreover, by considering continuous distributions, HSMMs can directly incorporate tracking data involving some cases with different time steps.

Overall the great flexibility of HSMMs makes them particularly attractive for the analysis of foraging movement patterns, since tracking data on animals are commonly available at high resolutions and are often acquired with irregular sampling rates [73].

### Real Behavioural Modes and the Relevance of Model Validation

The technological and methodological advances enable access to larger amounts of data and lead to continuously elaborating and applying new flexible modelling approaches for animal movement [73]. While following this trend, model validation and evaluation are often disregarded. [74] discuss this issue as a challenge in the future of statistics in general. It is also a challenge in movement modelling, particularly due to the conceptual and practical difficulties for obtaining groundtruthed data on animal behaviour.

Hence, when validating models with groundtruthed data, not only the models should be discussed but the data as well. In this work, we had access to a groundtruthed dataset, where behavioural modes were not chosen by us. Instead, they were previously defined by the predators themselves (fishermen) together with the on-board observers. This meant that states were not chosen in a way that they would be *a priori* easily recognizable (based on path geometry). On the other hand, it gave a great opportunity for evaluating the models performance for inferring real and complex behavioural mode sequences.

We reported 80% of global accuracy and 77%, 67% and 89% of F1 for fishing, searching and cruising, respectively, using the fitted HSMM model. Whereas the general performance is satisfactory, the searching mode appears difficult to identify. It might be explained by the nature of this behavioural mode. Interviewed fishermen anticipated that geometrical patterns in their tracks related to searching might vary greatly depending on several factors, especially whether or not they presume the inspected zone to be of high prey density. According to the fishermen, observed patterns for fishing and cruising are more stable. The low F1 score for searching may also be due to the time resolution of the data. As for fishing, the activity lasts ∼2 hours on average. However, 30-minute searching modes between two fishing modes were also reported by on-board observers. Such short state segments result in mixed signatures at the one-hour steps of the VMS data and can hardly be analysed. Higher-resolution tracking data should clearly contribute to a better identification of such searching modes, and would decrease the confusion rates with fishing and cruising; thus improve the inference accuracy of all behavioural modes. Moreover, as shown by the simulation study, HSMMs would increase their inference power if data resolution increases.

### Beyond Validation: Inference in Supervised and Semi-supervised Contexts

In supervised contexts, inferring behavioural modes is not only useful for achieving model validation. Supervised contexts do not necessarily imply that groundtruthed data on the behavioural modes of the whole population of tracks are available. Known behavioural modes may only be available for a subset of the tracks. In the case of fishermen, for instance, there may not be enough resources for on-board observers to register activities from all fishing trips of the entire population of vessels with tracking devices. For the Peruvian anchovy fishing fleet, more than 30000 fishing trips are tracked by VMS per year, but behavioural modes of only ∼300 of those trips are registered by on-board observers. Likewise, financial limitations could make possible tagging more individuals with GPS than with time-depth recorders (e.g. [9], [30]). Other limitations such as the memory card capacity for video-camera devices or daily diaries (e.g. [33]–[35]) may enable access to behavioural modes only at the beginning of the tracks. For all those cases, models trained and validated over the groundtruthed samples could be used for inferring behavioural modes over the remaining tracks or segments of tracks.

On the other hand, the non-supervised observed data could be used for updating the trained and validated models. In the machine learning domain, this is generally referred to as a semi-supervised setting. During the last years, numerous semi-supervised strategies have been proposed (see [37] for an extensive classification and revision). Among them, Markovian models naturally extend from the supervised case to the semi-supervised one, using the EM algorithm [75]. This appears as a particularly promising research direction for ecological studies, including the estimation of the resources (i.e. number of on-board observers, animal-borne electronic devices and analyses) to be allocated for gathering an optimal groundtruthed dataset.

### Modelling Extensions for Improving Inference Power

We have shown and discussed the advantages of Markovian models for taking into account the sequential nature of the data, while discriminative models typically achieve an independent inference of each state. Introducing past information on the observed variables may improve the inference performance of the discriminative models. We tested this possibility by introducing the immediate past values of the observed variables as new observed variables for the discriminative models. That meant adding four observed variables: speed at the previous step (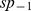), heading at the previous step (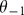), change of speed between the two previous steps (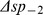) and turning angle between the two previous steps (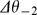). The immediate past values of 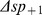 and 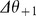 are 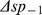 and 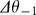, respectively. As indicated in the Methods section, for each model, from all the possible combinations of observed variables, we retained the subset of variables giving the greatest accuracy rate. Only for ANNs, a different subset of variables (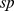,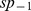,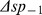,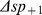,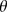,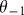,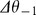,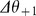) gave a higher accuracy. The new subset of observed variables involves the subset of variables from Table 3 plus four more observed variables. It improves inference of cruising modes (+0.5% in F1) and the general accuracy of the ANN model (+0.3%), although it decreases the performance over fishing and searching modes (−0.8% and −0.9% in F1, respectively).

Of course, more memory (past and future) in the observed variables could be added. But then, we would come across with the same memory-order dilemma than the one discussed for states in HMMs. Moreover, when we consider 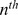 order past (or future) of an observed variable, the first (or last) 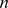 records will have missing values. This could be particularly annoying for classification using discriminative models. Another possibility would be to incorporate binary probabilities of the past states (i.e. presence or absence of a behavioural mode in the past states) for incremental training of discriminative models [76]. Incremental training involves training the model one time-step at a time, updating the model at each step. Nonetheless, this may result in over-fitting and large generalization errors. Besides, the direct application of this strategy may lead to drift effect. It means that inference at time 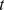 may be biased as it is driven by the effect of the inference at time 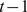. By contrast, Markovian models rely on a global inference, i.e. retrieving the state sequence that maximizes the posterior likelihood given the observed series. This global inference involves a forward-backward procedure which guarantees that the inference of any given state equally depends on past and future features along the trajectory.

Hence, combining the Markovian setting, which accounts for the sequential nature of the states, and the discriminative setting, which can achieve improved classification performance in high-dimensional non-Gaussian observation spaces, seems highly appealing. Such hybrid models have been investigated for different applications, especially speech recognition (e.g.[77]–[80]). They are stated as Markovian models that rely on the definition of an observation likelihood from the output of the chosen discriminative model (e.g. the discrimination SVM function for hybrid SVM-Markov models; [79]). However, the parametrization of the observation likelihood and the training of the hybrid model remain complex issues, which should be investigated in a future work.

Another attractive extension would be to model the observation process at the segment scale, i.e. at the same scale than that of the semi-Markov state process. That way, at each segment, one observation feature would be related to one state segment, which at the same time, would depend on the immediately preceding state segment. This modelling approach presents some potential advantages: it would imply modelling at the behavioural mode scale not only the state process but the observation process as well, and it could significantly improve the robustness to the presence of low-informative observation features.

The incorporation of informative priors could also play an important role in improving behavioural mode inference. For instance, predators may know *a priori* that the probability of foraging success increases/decreases with daylight. Since this knowledge affects their behaviour, hour-dependent state transition priors can be incorporated to the model. Likewise, priors on competition/association, as well as local climate conditions restricting mode transitions and durations could also be introduced in the model.

### Synthesis

We have shown a pioneer evaluation and comparison of Markovian and discriminative models for inferring behavioural modes within movement tracks in a supervised framework. The surpassing performance of Markovian models over the discriminative models highlights the importance of modelling state dynamics for accurately inferring the behavioural mode sequences. HMMs have been the most common approach in movement ecology. However, semi-Markov processes represent better the behavioural mode sequences than first-order Markov processes, since they explicitly model state duration and consider transitions at a segment scale. The HSMM performance on the groundtruthed dataset is slightly better than that of the HMM. As discussed above, this result responds to the nature of these particular behavioural modes as well as to the low resolution of the data. The ∼1 hour time steps are slightly below the characteristic durations of fishing and searching segments. Hence, regarding time steps, it is a favourable scenario for HMM. Through a simulation experiment, it was shown that increasing time resolution may decrease the accuracy obtained with HMMs and conversely increase the accuracy of HSMM inference. In foraging movement analysis, where (1) each type of behaviour contained in a track is typically characterized by a distinct duration, (2) tracking data are increasingly available at high resolutions, and (3) irregularity in sampling rates is not uncommon, we highly recommend the use of HSMMs. In addition, this work opens perspectives on the use of hybrid HSMM-discriminative models, where a discriminative setting for the observation process of HSMMs could greatly improve inference performance.

## Supporting Information

Figure S1 **Example of a sequence with its real and inferred behavioural modes.**1’s and 0’s in recall/precision represent a positive or null recall/precision corresponding to each behavioural mode, respectively. C = cruising, S = searching and F = fishing.
(TIF)

Table S1 **Distributions with significant fits under Cramér von Mises test for each observed variable and duration conditioned on states, for the HSMM in** **Table 3** **of the manuscript.** AIC values are in parentheses.
(DOC)

Text File S1 **Details on calculations of accuracy, precision, recall and F1 indicators.**
(DOC)

Text File S2 **Details on the simulation study.**
(DOC)

Text File S3 **Matlab™ code for HSMM Viterbi algorithm.**
(TXT)
