# Supplementary figures and images for: Hidden Markov Models: The Best Models for Forager Movements?
Source: PLoS One. 2013 Aug 23;8(8):e71246. doi: 10.1371/journal.pone.0071246 (PMC3751962; doi:10.1371/journal.pone.0071246)

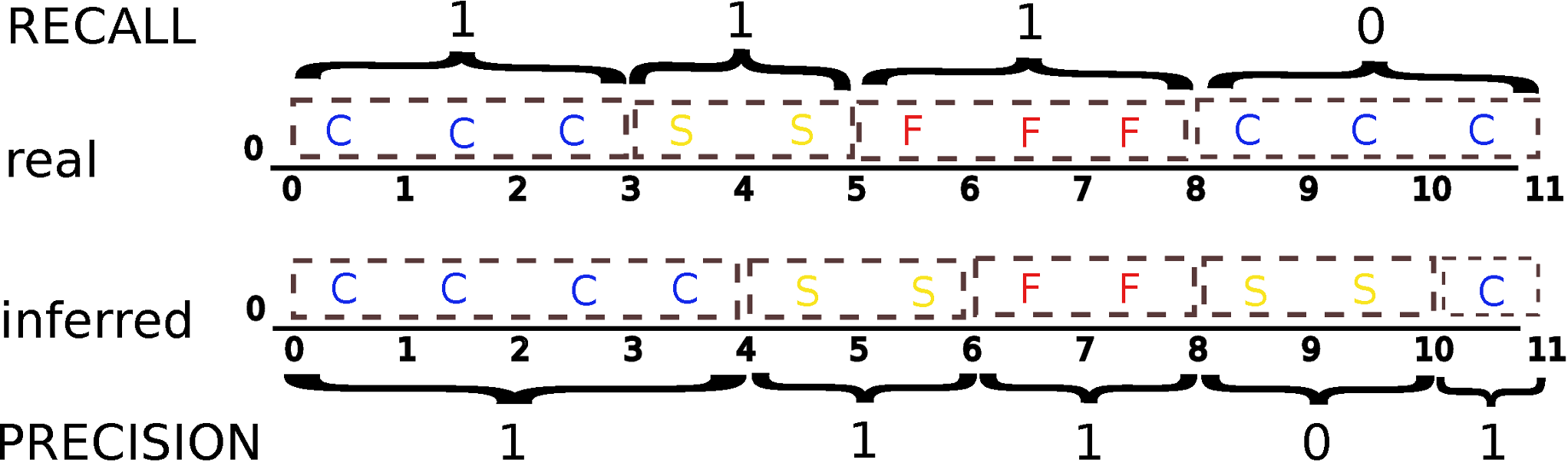

Supplement: Figure S1 — Example of a sequence with its real and inferred behavioural modes.1’s and 0’s in recall/precision represent a positive or null recall/precision corresponding to each behavioural mode, respectively. C = cruising, S = searching and F = fishing. (TIF) [file pone.0071246.s001.tif]
