# Supplementary material for: Hidden Markov Models: The Best Models for Forager Movements?
Source: PLoS One. 2013 Aug 23;8(8):e71246. doi: 10.1371/journal.pone.0071246 (PMC3751962; doi:10.1371/journal.pone.0071246)
Supplement: Table S1 — Distributions with significant fits under Cramér von Mises test for each observed variable and duration conditioned on states, for the HSMM in Table 3 of the manuscript. AIC values are in parentheses. (DOC) [file pone.0071246.s002.doc]

Supporting Information Table S1. Distributions with significant fits under Cramér von Mises test for each observed variable and duration conditioned on states, for the HSMM in Table 3 of the manuscript. AIC values are in parentheses.

| Observed Variable | Searching | Fishing | Cruising |
| --- | --- | --- | --- |
| sp | Generalized Pareto (4944.7) | Generalized extreme value (1451.6) | Gaussian mixture (6849.6) |
| Δsp_+1_ | Laplace (5179.2)  Normal (5203.5)  Truncated normal (5207.4) | Gumbel (4432.2) | Student’s t (6849.6) |
| Duration | Generalized extreme value (1045.2) | Lognormal (784.9) | Generalized extreme value (1182.0)  Lognormal (1206.8)  Logistic (1226.7) |
