# Supplementary material for: Hidden Markov Models: The Best Models for Forager Movements?
Source: PLoS One. 2013 Aug 23;8(8):e71246. doi: 10.1371/journal.pone.0071246 (PMC3751962; doi:10.1371/journal.pone.0071246)
Supplement: Text File S1 — Details on calculations of accuracy, precision, recall and F1 indicators. (DOC) [file pone.0071246.s003.doc]

Supporting Information Text File S1. Details on calculations of accuracy, precision, recall and F1 indicators.

Fig. S1 illustrates a sequence starting at time 0 and ending at 11, where the true behavioural modes are given and compared with the inferred ones. Accuracy is the percentage of steps where the inferred states correspond to the real ones, so it is equal to 7/11 x 100 = 63.6%.

Precision is the percentage of inferred segments where the inferred behavioural mode corresponds to the true one. An inferred mode *m* starting at time *t* and ending at time *t+u* is said to correspond to a true mode if there is a true mode *m* in the time interval *[t,t+u]* with an associated duration of at least *u/2*. The *u/2* threshold responds to the limitations of the time resolution of the sequence (~1 per hour) and the mean duration of the modes (~2 hours for fishing and searching). Precision, as well as recall and F1, are computed individually for each behavioural mode.

In the example of Fig. S1, there are two inferred segments for cruising. According to the definition above, the two inferred cruising segments correspond to true cruising segments. Thus,

Precision(cruising) = (1+1)/2 x 100 = 100%

Likewise, for searching and fishing we have:

Precision(searching) = 1/2 x 100 = 50%

Precision(fishing) = 1/1 x 100 = 100%

Recall is the percentage of real segments where the true mode is correctly inferred. A true mode *m* starting at time *t* and ending at time *t+u* is said to be correctly inferred if within the time interval *[t,t+u]* a behavioural mode *m* has been inferred with an associated duration of at least *u/2*. According to this definition, we compute for our example:

Recall(cruising) = 1/2 x 100 = 50%

Recall(searching) = 1/1 x 100 = 100%

Recall(fishing) = 1/1 x 100 = 100%

Since F1 is defined as the harmonic mean of recall and precision, we get:

F1(cruising) = 0.667

F1(searching) = 0.667

F1(fishing) = 1
