# Supplementary material for: Hidden Markov Models: The Best Models for Forager Movements?
Source: PLoS One. 2013 Aug 23;8(8):e71246. doi: 10.1371/journal.pone.0071246 (PMC3751962; doi:10.1371/journal.pone.0071246)
Supplement: Text File S2 — Details on the simulation study. (DOC) [file pone.0071246.s004.doc]

Supporting Information Text File S2. Details on the simulation study.

We consider only one observed variable in our simulation, in order to keep it simple. The probability distribution of this variable conditioned on one state is Gaussian, with μ=11 and σ=3. Its distribution conditioned on the other state is truncated normal with μ=6 and σ=5, and bounded on [0,25].

The one-second rate state sequence is simulated considering a logistic distribution with parameters μ=6.89 and *s*=1.21 for the duration of one state, and a generalized extreme value (GEV) distribution with parameters ξ=0.37, σ=0.4 and μ=2.1 for the duration of the other state. For the groundtruthed data study, logistic and GEV distributions are also used for modelling the duration of two of the behavioural modes.
